# Mortality Table

**Published:** 1879-12

**Authors:** 


### MORTALITY TABLE.

Condensed from National Board of Health Bulletin for the four weeks ending Nov. 1st, 1879.

| CITIES. | Estimated Population. | Deaths. | Death rate per 1000. |
|---|---|---|---|
| Baltimore, - - - - - | 400,000 | 517 | 16.80 |
| Boston, - - - - - | 365,000 | 525 | 18.42 |
| **BUFFALO,** - - - - | **170,000** | **160** | **11.79** |
| Chicago, - - - - - | 460,000 | 661 | 18.68 |
| Cleveland, - - - - - | 160,000 | 206 | 16.70 |
| Louisville, - - - - - | 175,000 | †80 | 11.32 |
| New York, - - - - - | 1,097,000 | 1911 | 22.64 |
| Philadelphia, - - - - | 901,000 | 927 | 13.37 |
| Rochester, - - - - - | 90,000 | *81 | 15.57 |
| St. Louis, - - - - - | 500,000 | 380 | 9.88 |

† Only two weeks reported. * Only three weeks reported to National Board of Health.

